# Real world evidence of the association between medication and life expectancy in elderly inflammatory bowel disease: a population-based cohort study

**DOI:** 10.1186/s12876-021-02083-y

**Published:** 2022-01-04

**Authors:** M. Ellen Kuenzig, Douglas G. Manuel, Jessy Donelle, Eric I. Benchimol

**Affiliations:** 1 Division of Gastroenterology, Hepatology and Nutrition, Children’s Hospital of Eastern Ontario (CHEO) Inflammatory Bowel Disease Centre, Children’s Hospital of Eastern Ontario, Ottawa, ON Canada; 2 CHEO Research Institute, Ottawa, ON Canada; 3 ICES uOttawa, Ottawa, ON Canada; 4 Division of Gastroenterology, Hepatology and Nutrition, SickKids Inflammatory Bowel Disease Centre, The Hospital for Sick Children, 555 University Avenue, Toronto, ON M5G 1X8 Canada; 5 Child Health Evaluative Sciences, SickKids Research Institute, Toronto, ON Canada; 6 Ottawa Hospital Research Institute, Ottawa, ON Canada; 7 School of Epidemiology and Public Health, University of Ottawa, Ottawa, ON Canada; 8 Department of Paediatrics and Institute of Health Policy, Management and Evaluation, University of Toronto, Toronto, ON Canada

**Keywords:** Life expectancy, Medication, Inflammatory bowel disease, Elderly, Mortality, Pharmacoepidemiology, Health administrative data

## Abstract

**Background:**

Life expectancy in people with inflammatory bowel disease (IBD) has increased but remains shorter than in people without IBD. We describe the life expectancy associated with IBD therapies among the growing number of older adults living with IBD.

**Methods:**

Older adults (≥ 65 years) with IBD were identified from population-based health administrative data using a validated algorithm. Life expectancy on patients’ 65th birthday, stratified by sex, was calculated using a period life table approach from age- and sex-specific mortality rates among patients receiving immunomodulator monotherapy, biologic monotherapy, combination therapy, mesalamine, systemic steroids, and no therapy.

**Results:**

Among 28,260 older adults with IBD (239,125 person-years of follow-up), life expectancy at 65 years was longest for patients taking mesalamine (females: 22.1 years, 95% CI 21.8–22.5; males: 19.6 years, 95% CI 19.3–20.0) and shortest for patients taking steroids (females: 11.7 years, 95% CI 11.0–12.4; males 10.3 years, 95% CI 9.7–10.8). Life expectancy was similar for patients receiving immunomodulator monotherapy and biologic monotherapy. Immunomodulator monotherapy was associated with a reduction in life expectancy compared to combination therapy by 5.1 (95% CI 2.3–7.8) in females and 2.8 years (95% CI 0.1–5.5) in males.

**Conclusions:**

Life expectancy varies across therapies used for IBD, with differences likely arising from a combination of medication effectiveness, safety profiles, disease severity, and comorbid conditions. These considerations should be balanced when deciding on a therapeutic approach for the management of IBD in older adults.

**Supplementary Information:**

The online version contains supplementary material available at 10.1186/s12876-021-02083-y.

## Introduction

Inflammatory bowel disease (IBD), comprised of subtypes Crohn’s disease and ulcerative colitis, is a chronic immune-mediated inflammatory condition primarily affecting a patient’s gastrointestinal tract. People with IBD often experience a relapsing and remitting disease course. Over the past three decades, the armamentarium of therapies available to treat IBD has evolved greatly. This has been accompanied by other improvements in patient care, including better access to specialist care and treatment strategies (e.g., the treat-to-target approach and therapeutic drug monitoring). Since the introduction of biologic therapy for IBD, life expectancy among people with IBD has increased [1]. However, it remains shorter than in those without IBD.

Although IBD is most often diagnosed in adolescents and young adults, the prevalence of IBD is rising fastest among seniors [2]. The reasons for this increase are two-fold. First, the populations of industrialized countries (with historically high rates of IBD) are aging. As the general population ages, so does the population of IBD patients. Simultaneously, there are a growing number of new diagnoses made in older people [3].

Managing IBD in the elderly is complicated by the presence of age-related comorbidities such as cardiovascular disease and diabetes. Further, biologic therapies are associated with an increased risk of infections and malignancy, particularly among seniors [4–6]. Thus, it is imperative that older IBD patients receive care that not only reduces the burden of their IBD, but also maximizes their longevity. We describe the life expectancy and mortality associated with different IBD therapies among older adults.

## Methods

### Data sources

We conducted a population-based cohort study using health administrative data from Ontario, Canada. These data include information on all contacts with the health care system for all residents of Ontario who qualify for universal single-payer healthcare (> 99% of the population). Hospitalizations are identified using the Canadian Institute for Health Information’s (CIHI) Discharge Abstract Database. Emergency department visits are identified using CIHI’s National Ambulatory Care Reporting System. Outpatient visits are identified through Ontario Health Insurance Plan physician billing data required for physician renumeration. These health administrative databases are linked deterministically using a unique, encrypted health identification number to the Registered Persons Database (demographic characteristics, including start and end dates of provincial health coverage eligibility), Vital Statistics (date of death), and the Ontario Drug Benefit database. The Ontario Drug Benefit database includes data on all outpatient prescriptions among Ontario residents ≥ 65 years of age, including the type of medication prescribed, the dose of the medication, and the total number of days the medication was prescribed for. Data are maintained by ICES via an agreement with the Ontario Ministry of Health (MOH) and Ministry of Long-Term Care (MLTC), with the full database available to researchers in an uncleaned and unedited format [7]. ICES is an independent, non-profit research institute whose legal status under Ontario’s health information privacy law allows it to collect and analyze health care and demographic data, without consent, for health system evaluation and improvement.

### Study population

Elderly patients ≥ 65 years with IBD were identified from Ontario health administrative data using previously-validated age-specific algorithms [8]. IBD patients who did not turn 65 years old during the study period were excluded. Individuals diagnosed prior to age 65 required at least 5 outpatient visits or hospitalizations with an International Classification of Diseases (ICD)-9 (555, 556) or ICD-10 code (K50, K51) for IBD within 4 years (sensitivity 76.8–92.3%; specificity 96.2–99.1%; positive predictive value 81.4%; negative predictive value 95.0%) and those diagnosed ≥ 65 years additionally required a prescription for an IBD medication (sensitivity 59.3–78.3%; specificity 98.2–99.0%, positive predictive value 71.1%, negative predictive value 98.3%). We differentiated between subtypes Crohn’s disease and ulcerative colitis based on the last 5 of 9 outpatient visits, with an accuracy of 91.1% noted in validation studies [8].

### Study design

We conducted a retrospective cohort study of seniors with IBD who were ≥ 65 years of age between July 1, 1997 and June 30, 2017. Patients younger than 65 at diagnosis contributed person-time to the study beginning on their 65th birthday. When patients were diagnosed ≥ 65 years, they began contributing data on their date of IBD diagnosis (the date with their first health care encounter with an associated diagnosis of IBD). Patients were followed until death, migration out of Ontario, or the end of the follow-up period (June 30, 2017).

We compared life expectancy among individuals receiving the following medications: (1) monotherapy with an immunomodulator (azathioprine, 6-mercaptopurine or methotrexate); (2) biologic monotherapy; (3) combination therapy with a biologic and an immunomodulator; (4) mesalamine; (5) systemic steroids; and (6) no therapy. Life expectancy was calculated separately for males and females due to known differences in life expectancy among males and females in the general population. People with IBD ≥ 65 years contributed to the exposure period for the duration they were on a medication based on prescriptions identified in the Ontario Drug Benefit database. Additional file 1: Table S1 provides a list of all medications and their drug identification numbers (DINs) used in this study. They were assigned to an age group (65–69, 70–74, 75–80, 85–90, and 90+) based on their age when each prescription was dispensed. Patients were assigned to a no-therapy group for time periods when they had no active prescriptions. For non-biologic medications, the duration of therapy was determined from the number of days supplied in the Ontario Drug Benefit database. A 30-day window was allowed between the end of prescriptions for non-biologic medication and the next prescription to account for potential non-adherence. We used a 12-week window for biologics typically administered every 8 weeks (infliximab, ustekinumab, and vedolizumab) and a 6-week window for all other biologics (adalimumab, golimumab, certolizumab, and natalizumab). Patients on combination therapy received a prescription for an immunomodulator and a biologic within 3 months of each other. Otherwise, these patients could contribute person-time to biologic or immunomodulator monotherapy groups. All other groups were non-mutually exclusive, meaning that patients could contribute person time to multiple medication groups simultaneously.

### Statistical analysis

We calculated sex-specific mortality rates for each medication in each of the following age groups: 65–69, 70–74, 75–79, 80–84, 85–89, and 90+. These mortality rates were then used to calculate life expectancy at age 65 with a period life-table approach, assuming that age- and sex-specific mortality rates for a specific time period remain constant over a person’s life [9]. Hsieh’s modification was used for the last age group [10]. This approach applies age- and sex-specific mortality rates to a hypothetical cohort of individuals, beginning at age 65, and assumes that these mortality rates remain constant over time [9]. This hypothetical cohort has no underlying characteristics prior to being subjected to the mortality rates associated with each medication. Period life expectancy transforms mortality rates into a measure of longevity, increasing interpretability [11].

We determined the differences between the life expectancy at age 65, comparing patients in each medication group, stratified by sex. The impact of medication on life expectancy was explored in (1) people with IBD; (2) people with Crohn’s disease; and (3) people with ulcerative colitis. Differences were considered statistically significant when the 95% confidence interval (CI) did not include the null value of 0. We plotted survival curves based on the expected proportion of people remaining alive in each age interval for each medication group, beginning at 65 years of age.

We also used age- and sex-specific mortality rates and the age- and sex-specific distribution of prevalent IBD cases ≥ 65 years of age on July 1, 2016 to determine age-standardized sex-specific mortality rates for each medication group. Due to the small number of deaths in some medication groups, mortality rates are only presented for overall IBD.

Statistical analyses were conducted using SAS Version 9.4 (SAS Institute, Cary, NC, U.S.A.).

#### Sensitivity analysis

To estimate the influence of post-operative mortality on life expectancy estimates, we conducted a sensitivity analysis where individuals requiring surgery were censored at the time of surgery. We identified individuals requiring surgery using previously validated procedural codes for small bowel resection (Crohn’s disease only) and colectomy (Crohn’s disease and ulcerative colitis) (Additional file 1: Table S2) [12, 13]. We repeated disease-specific life expectancy calculations and determined the difference in life expectancy across medication groups. All analyses were stratified by IBD type due to differences in surgical procedures across IBD types.

## Results

A total of 28,260 people with IBD with 239,125 person-years of follow-up were included in the study (Table 1).

**Table 1:** Characteristics of individuals included in the study

	N (%)	Total person-years of follow-up
Total number of cases included in analysis	28,260	239,125.95
Female	14,678 (51.9)	
Age at IBD diagnosis		
Diagnosed ≤ 65 years	17,662 (62.5)	
Diagnosed ≥ 65 years	5554 (19.7)	
Unknown^a^	5044 (17.9)	
Rural residence^b^	4344 (15.4)	
Mean neighbourhood income quintile		
Q1	4678 (16.7)	
Q2	5420 (19.4)	
Q3	5642 (20.2)	
Q4	5662 (20.3)	
Q5	6547 (23.4)	
Drug group		
Immunomodulator Monotherapy	4789 (16.9)	12,141.96
Biologic Monotherapy	1381 (4.9)	3010.39
Combination therapy	844 (3.0)	1764.27
Mesalamine	17,625 (62.4)	81,128.99
No therapy	28,260 (100.0)	95,492.74
Systemic steroids	14,647 (51.8)	12,220.14
Type of IBD		
Crohn’s disease	10,870 (38.5)	n/a
Ulcerative colitis	15,992 (56.6)	n/a
IBD-type unclassified	1398 (4.9)	n/a

*IBD* inflammatory bowel disease

^a^First diagnostic code for IBD occurred ≥ 65 years of age but did not have an 8-year washout period prior to first IBD code

^b^At first diagnostic code for IBD

### Life expectancy

**Fig. 1:**
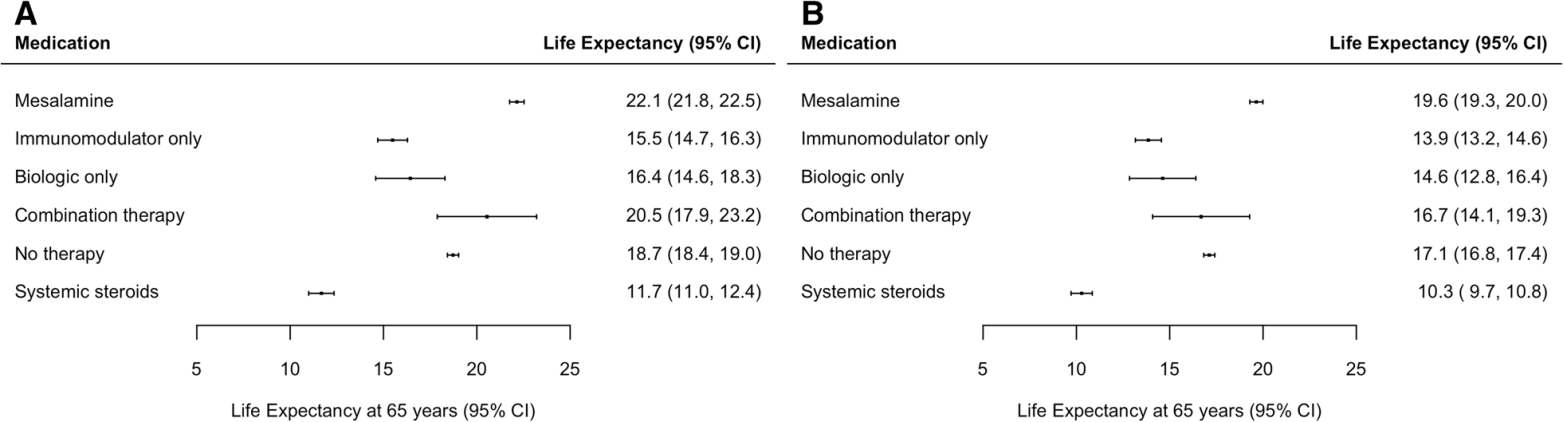
Life expectancy at 65 years of age in (**A**) females and (**B**) males with inflammatory bowel disease, stratified by type of medical treatment

**Fig. 2:**
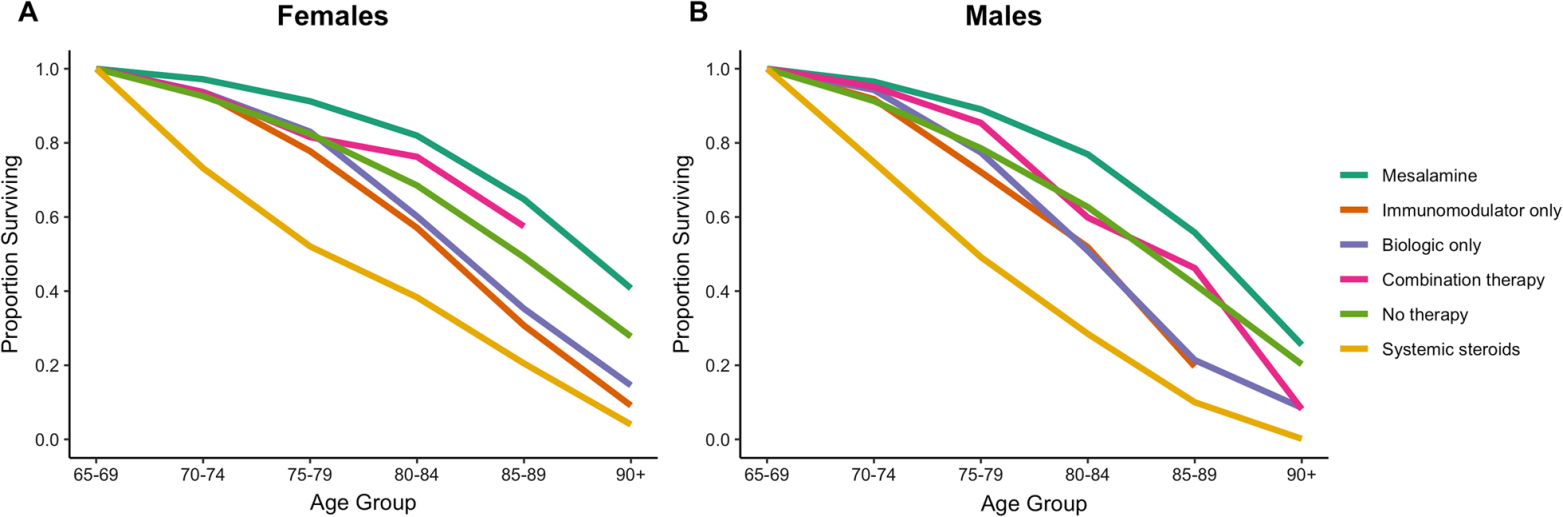
The proportion of people expected to be alive at each age interval based on type of medical treatment in (**A**) females and (**B**) males with inflammatory bowel disease

The life expectancy at age 65 years for patients receiving mesalamine was 22.1 years (95% CI 21.8–22.5) for females and 19.6 years (95% CI 19.3–20.0) for males (Fig. 1). This life expectancy was significantly longer when compared with the life expectancy of patients on any other treatment, with the exception of females on combination therapy with a biologic and immunomodulator (Table 2; Fig. 2). Life expectancy was 3.4 (95% CI 2.9–3.9) years longer in females and 2.5 years (95% CI 2.1–3.0) years longer in males on mesalamine when compared to patients in the no-therapy group. In patients with Crohn’s disease, the difference between mesalamine and combination therapy was not statistically significant (Additional file 1: Table S3 and Additional file 1: Figs. S1 and S2). Mesalamine was associated with longer life expectancy compared with all other medications in females with ulcerative colitis and in most other medications in males with ulcerative colitis (Additional file 1: Table S4 and Additional file 1: Figs. S3A and S4A). In males with ulcerative colitis, biologic monotherapy was the only medication not associated with a significantly different life expectancy relative to mesalamine (Additional file 1: Table S4 and Additional file 1: Figs. S3B and S4B).

**Table 2:** Differences in life expectancy at 65 years comparing medications used to treat seniors with inflammatory bowel disease

	Mesalamine	Immunomodulator monotherapy	Biologic monotherapy	Combination therapy	Systemic steroids
No therapy					
Females	**− 3.4 (− 3.9, − 2.9)**	**3.2 (2.4, 4.1)**	**2.3 (0.4, 4.2)**	− 1.8 (− 4.5, 0.9)	**7.1 (6.3, 7.8)**
Males	**− 2.5 (− 3.0, − 2.1)**	**3.3 (2.5, 4.0)**	**2.5 (0.7, 4.3)**	0.4 (− 2.2, 3.1)	**6.8 (6.2, 7.5)**
Mesalamine					
Females		**6.7 (5.8, 7.5)**	**5.7 (3.8, 7.6)**	1.6 (− 1.1, 4.3)	**10.5 (9.7, 11.3)**
Males		**5.8 (5.0, 6.6)**	**5.0 (3.2, 6.8)**	**3.0 (0.3, 5.6)**	**9.4 (8.7, 10.0)**
Immunomodulator monotherapy					
Females			− 0.9 (− 3.0, 1.1)	**− 5.1 (− 7.8, − 2.3)**	**3.8 (2.8, 4.9)**
Males			− 0.8 (− 2.7, 1.1)	**− 2.8 (− 5.5, − 0.1)**	**3.6 (2.7, 4.5)**
Biologic monotherapy					
Females				**− 4.1 (− 7.4, − 0.9)**	**4.8 (2.8, 6.7)**
Males				− 2.1 (− 5.2, 1.1)	**4.3 (2.5, 6.2)**
Combination therapy					
Females					**8.9 (6.1, 11.6)**
Males					**6.4 (3.7, 9.1)**

Differences correspond to the medication referenced in the column name subtracted from the medication reference in the row name (i.e., LE_row _– LE_column_). Significant differences are indicated in bold font

Comparing patients on immunomodulator monotherapy and biologic monotherapy, there was no statistically significant difference in life expectancy (differences presented as life expectancy with biologic monotherapy subtracted from immunomodulator therapy; females: ∆-0.9 years (y), 95% CI − 3.0 to 1.1; males: ∆-0.8y, 95% CI  − 2.7 to 1.1) (Table 2). This was consistent in males and females with Crohn’s disease (Additional file 1: Table S3) and females with ulcerative colitis (Additional file 1: Table S4). In males with ulcerative colitis, immunomodulator monotherapy was associated with a decrease of 3.7 years (95% CI 0.3–7.1) in life expectancy compared with biologic monotherapy.

Comparing patients on immunomodulator monotherapy to those on combination therapy with a biologic, there was a reduction in life expectancy by 5.1 years (95% CI 2.3–7.8) in females and 2.8 years (95% CI 0.1–5.5) in males (Table 2). This association persisted in females with Crohn’s disease but was not statistically significant in males with Crohn’s disease, or in males and females with ulcerative colitis (Additional file 1: Tables S3 and S4).

Females on biologic monotherapy had a shorter life expectancy than those on combination therapy (Table 2). This association persisted for females with Crohn’s disease (Additional file 1: Table S3) but was not different in males with any type of IBD or females with ulcerative colitis (Table 2, Additional file 1: Tables S3 and S4).

In patients taking systemic steroids, life expectancy at age 65 years was 11.7y (95% CI 11.0–12.4y) for females and 10.3y (95% CI 9.7–10.8y) for males (Fig. 1). Treatment with systemic steroids was associated with significantly decreased life expectancy when compared to all other treatments for females and all treatments exception combination therapy for males (Table 2; Fig. 2). Life expectancy was significantly reduced in patients on steroids for Crohn’s disease and for almost all comparisons in patients with ulcerative colitis (Additional file 1: Tables S3 and S4).

### Mortality

**Fig. 3:**
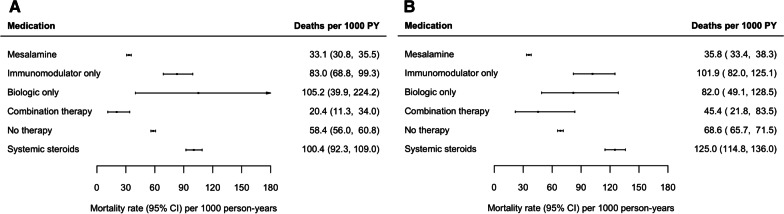
Age-standardized mortality rates among (**A**) females and (**B**) males with inflammatory bowel disease, stratified by medication use

In females, the lowest age-standardized mortality rate was observed among patients receiving combination therapy with a biologic and immunomodulator (20.4 deaths per 1000 PY, 95% CI 11.3–34.0), followed by mesalamine (33.1 deaths per 1000 PY, 95% CI 30.8–35.5; Fig. 3A). The highest mortality rates were observed among those receiving systemic steroids (100.4 deaths per 1000 PY, 95% CI 92.3–109.0) and biologic monotherapy (105.2 deaths per 1000 PY, 95% CI 39.9–224.2). Mortality rates specific to individuals with Crohn’s disease and ulcerative colitis could not be reported due to the small number of events.

In males, the lowest age-standardized mortality rate was observed among patients receiving mesalamine (35.8 deaths per 1000 PY, 95% CI 33.4–38.3) followed by the no-therapy group (45.2 deaths per 1000 PY, 95% CI 43.3–47.2) and combination therapy with a biologic and an immunomodulator (45.4 deaths per 1000 PY, 95% CI 21.8–83.5; Fig. 3B). The highest mortality rate was observed among those receiving systemic steroids (125.0 deaths per 1000 PY, 95% CI 114.8–136.0). Mortality rates specific to individuals with Crohn’s disease and ulcerative colitis could not be reported due to the small number of events.

### Sensitivity analysis: life expectancy censoring patients at surgery

Censoring patients at surgery resulted in slightly decreased life expectancy across all medication groups (Additional file 1: Figs. S5 and S6). Life expectancy continued to be the longest among those receiving mesalamine and shortest among those receiving systemic steroids. The differences in life expectancy were identical in those with ulcerative colitis when compared to the primary analysis (Additional file 1: Tables S5 and S6; Additional file 1: Figs. S7 and S8). In Crohn’s disease, the differences in life expectancy across medication groups were generally similar compared to the main analyses, with some notable exceptions: (1) in males, the differences in life expectancy comparing biologic monotherapy with other therapies were much larger in magnitude and biologic monotherapy was associated with a significantly shorter life expectancy than immunomodulator monotherapy or combination therapy; (2) in females, life expectancy in patients receiving combination therapy was no longer significantly different than the no-therapy group or biologic therapy, although differences were similar in magnitude to the primary analysis.

## Discussion

Life expectancy at 65 years of age and mortality rates varied significantly in patients taking different classes of medications for IBD. Life expectancy at 65 was longest for patients receiving mesalamine: 22.1 years for females and 19.6 years for males. This means that a female with IBD on mesalamine could expect to live to 87.1 years of age, while a male with IBD on mesalamine could expect to live to 84.6 years of age. In contrast, life expectancy was shortest for patients receiving systemic steroids: 11.7 years for females and 10.3 years for males meaning that females and males taking systemic steroids could expect to live to 76.7 and 75.3 years of age, respectively. Age-standardized mortality rates were also highest among those receiving systemic steroids.

The longer life expectancy of people with IBD receiving mesalamine compared to any other medication group was likely due to its association with mild disease severity which unfortunately cannot be measured using health administrative data. However, it was reassuring that life expectancy was similar among people receiving immunomodulators and biologics, who presumably had moderate to severe disease activity at the time these medications were initiated. Mesalamine was previously associated with decreased mortality in ulcerative colitis, but not Crohn’s disease [14]. Our observed association between steroid use with life expectancy and mortality is consistent with previously-reported findings of elevated mortality patients with IBD [15–17]; however, not all studies have reported increased mortality among patients receiving steroids [14].

The relative impact of immunomodulators and biologics on mortality remains inconclusive, with some evidence suggesting higher mortality with biologics and others suggesting a similar risk of mortality across therapies [14, 16, 18]. Mortality was slightly decreased among people receiving combination therapy compared to biologic monotherapy [16]. In our study, combination therapy was associated with a significantly longer life expectancy than immunomodulator monotherapy and slightly longer life expectancy than biologic monotherapy.

Although steroids were associated with a shorter life expectancy and mesalamine was associated with longer life expectancy compared to other medications, these results may have been confounded by disease phenotype or severity, as markers of disease severity, extent, and phenotype are not available in health administrative data. People ≥ 65 years receiving biologic therapy likely had moderate or severe disease that was refractory to other therapies; however, their life expectancy was similar to patients receiving no medical therapy. This is reassuring since patients in the no-therapy group likely had a very mild disease course, implying that biologics resulted in a reduced risk of disease-related complications and resulting mortality. Further, our sensitivity analysis censoring patients at surgery indicates that post-operative mortality did not have a significant impact on the differences in life expectancy across therapy groups, especially among the no-therapy group. As well, people receiving mesalamine may have had more mild non-refractory disease which may have contributed to their longer life expectancy relative to other treatment groups. Life expectancy among people receiving steroids may be decreased due to refractory disease resulting in increased likelihood of using systemic corticosteroids, or risks associated with these medications. Steroids may also be used for non-IBD comorbid conditions with a high risk of death, or as part of palliative care. Therefore, the reduced life expectancy may have been due to IBD complications, steroid side effects, or is acting as a marker of comorbidities or end-of-life care.

Life expectancy was slightly decreased in all medication groups after censoring individuals requiring an IBD-related intestinal resection or colectomy. This decrease may have resulted from the shortened follow-up available in all medication groups (without an equivalent decrease in the number of deaths). Alternatively, patients requiring surgery may have been systematically different from individuals not requiring surgery and our findings may have been confounded by disease severity, comorbidities, or other factors not accounted for in our analysis.

The Ontario Drug Benefit database only includes complete population-level data on medications for people ≥ 65 years of age, limiting our ability to evaluate the impact of medications in younger patients with IBD. Because we only had medication data available for patients ≥ 65 years of age, we had relatively small number of patients on some therapies (e.g., combination therapy), limiting our power to determine statistical differences in the life expectancy associated with these therapies and we did not have a large enough sample size to evaluate the association between these therapies and post-operative mortality among patients requiring surgery. Further, despite changes in the frequency of biologics and the strategies used to effectively use these medications (e.g., therapeutic drug monitoring, treat-to-target), we were not able to evaluate the impact of these changes on life expectancy. As with all studies using health administrative data, our findings are subject to the risk of misclassification bias [19]. However, we used validated algorithms to identify patients with IBD and to classify them as having Crohn’s disease and ulcerative colitis [8]. In addition, in the absence of controlled comparative studies involving older adults with IBD, real-world pharmaco-epidemiology research using routinely collected health data is the most feasible way of identifying worrisome trends in the association between various medications and life expectancy.

## Conclusions

Life expectancy and mortality rates vary across therapies used in the management of IBD. These differences likely arise from a combination of medication effectiveness, safety profiles, disease severity, and comorbid conditions. When deciding on a therapeutic approach for the management of IBD in older adults, the impact of life expectancy and risk of mortality should be balanced with the importance of adequately treating active inflammation and reducing complication rates.

## Supplementary Information

**Additional file 1. Table S1**. List of drug identification numbers used to identify medications included in this study.** Table S2**. List of validated codes used to identify patients requiring small bowel resection (Crohn’s disease) or colectomy (Crohn’s disease and ulcerative colitis).** Table S3**. Differences in life expectancy at 65 years comparing medications used to treat Crohn’s disease in seniors. Differences correspond to the medication referenced in the column name subtracted from the medication reference in the row name (i.e., LErow – LEcolumn). Significant differences are indicated in bold font.** Table S4**. Differences in life expectancy at 65 years comparing medications used to treat ulcerative colitis in seniors. Differences correspond to the medication referenced in the column name subtracted from the medication reference in the row name (i.e., LErow – LEcolumn). Significant differences are indicated in bold font.** Table S5**. Differences in life expectancy at 65 years comparing medications used to treat Crohn’s disease in seniors, censoring at intestinal resection or colectomy. Differences correspond to the medication referenced in the column name subtracted from the medication reference in the row name (i.e., LErow – LEcolumn). Significant differences are indicated in bold font.** Table S6**. Differences in life expectancy at 65 years comparing medications used to treat ulcerative colitis in seniors, censoring at colectomy. Differences correspond to the medication referenced in the column name subtracted from the medication reference in the row name (i.e., LErow – LEcolumn). Significant differences are indicated in bold font.** Figure S1**. Life expectancy at 65 years of age in (A) females and (B) males with Crohn’s disease, stratified by type of medical treatment.** Figure S2**. The proportion of people expected to be alive at each age interval based on type of medical treatment in (A) females and (B) males with Crohn’s disease.** Figure S3**. Life expectancy at 65 years of age in (A) females and (B) males with ulcerative colitis, stratified by type of medical treatment.** Figure S4**. The proportion of people expected to be alive at each age interval based on type of medical treatment in (A) females and (B) males with ulcerative colitis.**Figure S5**. Life expectancy at 65 years of age in (A) females and (B) males with Crohn’s disease, stratified by type of medical treatment and censored at intestinal resection or surgery.** Figure S6**. Life expectancy at 65 years of age in (A) females and (B) males with ulcerative colitis, stratified by type of medical treatment and censored at colectomy.** Figure S7**. The proportion of people expected to be alive at each age interval based on type of medical treatment in (A) females and (B) males with Crohn’s disease, censored at intestinal resection or colectomy.** Figure S8**. The proportion of people expected to be alive at each age interval based on type of medical treatment in (A) females and (B) males with ulcerative colitis, censoring at colectomy.

## Data Availability

The data from this study is held securely in coded from at ICES. While data sharing agreements prohibit ICES from making the data set publicly available, access may be granted to those who meet prespecified criteria for confidential access, available at www.ices.on.ca/DAS. The full data set creation plan and underlying analytic code are available from the authors upon request, understanding that the programs may rely upon coding templates or macros that are unique to ICES.

## Abbreviations

CI: Confidence interval
CIHI: Canadian Institute for health information
DIN: Drug identification number
IBD: Inflammatory bowel disease
ICD: International classification of diseases
MLTC: Ministry of long-term care
MOH: Ministry of health
PY: Person-years
