# Supplementary material for: Real world evidence of the association between medication and life expectancy in elderly inflammatory bowel disease: a population-based cohort study
Source: BMC Gastroenterol. 2022 Jan 4;22:5. doi: 10.1186/s12876-021-02083-y (PMC8728958; doi:10.1186/s12876-021-02083-y)

**Additional file 1**

**Supplemental Table 1. List of drug identification numbers used to identify medications included in this study.**

| Medication | Drug Identification Number |
| --- | --- |
| **Immunomodulators** | |
| **Thiopurines** | |
| Azathioprine | 4596, 2231491, 2236799, 2236819, 2242907, 2343002, 337854, 903187, 2231575, 2235352, 2237291, 2242148, 2243371, 2244895, 2248843, 2341115, 2358573, 2360918, 22123236 |
| Mercaptopurine | 4723, 2415275, 9857458, 922303, 2238895, 99100697, 99100698, 99100811, 99100816, 99100873, 99100874, 99100875, 99100876 |
| **Other immunomodulators** | |
| Methotrexate | 2182777, 2182955, 14915, 2170698, 2182750, 2182963, 2244798, 2398427, 2099705, 9857520, 614343, 632619, 1907204, 1911457, 2170655, 2182971, 2419173, 2422166, 2422174, 2422182, 2422190, 2422204, 99506020 |
| Methotrexate disodium | 321397, 321400, 2170663, 2170671, 410195, 519286 |
| Methotrexate sodium | 2182947, 614335, 874132, 2171767, 614327, 2084481, 2089920, 2122170, 2161168, 2177110, 2182939, 2283697, 2304767, 2320029, 2320037, 2320045, 2320053, 2327236, 2333023, 2377624, 2417626, 2454750, 2454769, 2454777, 2454823, 2454831, 2454858, 2454866, 2454874, 9991119 |
| Cyclosporine | 755591, 755605, 1907182, 2150662, 2150670, 2150689, 2237671, 2242821, 2247073, 2247074, 9857097, 9857613, 9857614, 9857176, 9857184, 9857192, 2150697, 2244324, 9857129, 9857214, 9857206, 9857770, 593249, 593257, 950513, 950548, 950556, 950792, 950793, 950807, 950815, 950823, 990013, 2334763, 2334771, 2334798, 66123905, 66123942, 66123943, 66123944, 66123945, 66950521, 99100387 |
| Tacrolimus | 2175983, 2175991, 2243144, 2416824, 2416832, 2296462, 2296470, 2296489, 2331667, 2176009, 9851984, 9852662, 9854786, 903944, 950645, 950653, 960039, 960632, 960634, 66123987, 66990973 |
| **Biologics** | |
| Infliximab (Remicade) | 2244016, 9852956, 2419475, 903607, 904056, 950899, 2419483, 92099967, 92244016, 99101167 |
| Adalimumab (Humira) | 2258595, 9854785, 9857294, 9857326, 9857327, 903684, 2458349, 2458357, 97799756, 97799757, 99100385 |
| Certolizumab pegol (Cimzia) | 2331675 |
| Golimumab (Simponi) | 2413183, 2324776, 2324784, 2413175, 2417472 |
| Ustekinumab (Stelara) | 2320673, 2320681, 903860, 999746, 2320672, 2459671 |
| Vedolizumab (Entyvio) | 2436841 |
| Natalizumab (Tysabri) | 2286386 |
| **Systemic** **Steroids** | |
| 16438, 16446, 16462, 21695, 28096, 28185, 30600, 30619, 30627, 30635, 30643, 30651, 30678, 30740, 30759, 30767, 30910, 30929, 30988, 36129, 36366, 93629, 210188, 213624, 232378, 249963, 252417, 269026, 271373, 280437, 285471, 295094, 312770, 349100, 354309, 489158, 501050, 504416, 550957, 598194, 610623, 664227, 888230, 1934325, 1964070, 1964976, 1977547, 2152541, 2230619, 2240684, 2240687, 2245400, 2245406, 2245532, 2250055, 2261081, 9850236, 9852883, 9854537, 9857120, 9857797, 9857798, 9857799, 3603, 7668, 10197, 12211, 16241, 16268, 16276, 16470, 16527, 21679, 21687, 22500, 22519, 23833, 28118, 30739, 30791, 30961, 30989, 31003, 36137, 93602, 133426, 153710, 154008, 156876, 157880, 176834, 177571, 178926, 178934, 194573, 194980, 210692, 232092, 250325, 250376, 271381, 278912, 280852, 298301, 298328, 300373, 308455, 338656, 338664, 338672, 338680, 386847, 482293, 482307, 482919, 496219, 501069, 501131, 508586, 525286, 544817, 589194, 598542, 607517, 643122, 664200, 696617, 716715, 716855, 732885, 732893, 732907, 751863, 770759, 783900, 828912, 828920, 868426, 868434, 868442, 872520, 872539, 874582, 878618, 878626, 888206, 888214, 888222, 891983, 900044, 900516, 901397, 901425, 903205, 903213, 903296, 903531, 905577, 906689, 908223, 990137, 993610, 999108, 1934333, 1934341, 1962736, 1962744, 1962752, 1962760, 1964068, 1964968, 2016575, 2063697, 2063700, 2063719, 2063727, 2204266, 2204274, 2230210, 2230211, 2231893, 2231894, 2231895, 2232748, 2232750, 2237044, 2237045, 2237046, 2239534, 2240685, 2241229, 2244395, 2245407, 2245408, 2260298, 2260301, 2311267, 2367947, 2367955, 2367963, 2367971, 2378843, 2387743, 6623833, 19649760, 66123379, 66123732, 66123756, 66888095, 66896071, 66901352, 90090250, 99100485, 99100550, 99100988, 99503007, 99503008 | |
| **5-ASAs** | |
| Mesalamine/Mesalazine | 2112752, 2112760, 2513564, 2242146, 2112795, 2112809, 2153521, 2153548, 2153556, 2267217, 2297558, 1914030, 1997580, 2112787, 2171929, 2099675, 2099683, 784508, 709034, 752630, 881651, 894877, 1940384**,** 2399466, 634557, 784494, 813141, 881643, 887080, 887099, 890944, 908622, 914030, 940384, 1914049, 2112779, 2128233, 2128241, 2128268, 2128276, 2238931, 2238932, 2351463, 2028778, 2113058, 2182653, 2182661, 2182688, 2182696, 2182718, 2182726, 2265257, 2348063 |
| Olsalazine | 875848, 2063808, 2006413 |
| Sulfasalazine | 263869, 598461, 685933, 2064480, 445126, 598488, 685925, 2064472, 544442, 24856, 158526, 24864, 410640, 613568, 637769, 637777, 637785, 893692, 901394, 903342, 903449, 1999567, 2004658, 2004682, 2004690, 2064499, 2067986, 2129051, 22123261, 99101333, 99101334 |

**Supplemental Table 2. List of validated codes used to identify patients requiring small bowel resection (Crohn’s disease) or colectomy (Crohn’s disease and ulcerative colitis).**

| **CCP Codes for Resection/Colectomy in Crohn’s Disease** | |
| --- | --- |
| 5741 | Multiple segmental resection of small intestine |
| 5742 | Other partial resection of small intestine |
| 5743 | Total removal of small intestine |
| 575 | Partial excision of large intestine |
| 5751 | Multiple segmental resection of large intestine |
| 5753 | Right hemicolectomy |
| 5755 | Left hemicolectomy |
| 576 | Total colectomy |
| 5752 | Cecectomy |
| 5754 | Resection of transverse colon |
| 5756 | Sigmoidectomy |
| 5759 | Other partial excision of large intestine |
| **CCP Codes for Colectomy in Ulcerative Colitis** | |
| 575 | Partial excision of large intestine |
| 5751 | Multiple segmental resection of large intestine |
| 5753 | Right hemicolectomy |
| 5755 | Left hemicolectomy |
| 576 | Total colectomy |
| 5752 | Cecectomy |
| 5754 | Resection of transverse colon |
| 5756 | Sigmoidectomy |
| 5759 | Other partial excision of large intestine |
| **CCI Codes for Resection/Colectomy in Crohn’s Disease** | |
| 1NK87.x | Excision partial, small intestine |
| 1NM87.x | Excision partial, large intestine |
| 1NM89.x | Excision total, large intestine |
| 1NQ87.x | Excision partial, rectum |
| 1NQ89.x | Excision total, rectum (including proctocolectomy) |
| 1NQ90.x* | Excision total with reconstruction, rectum |
| 1NM91.x** | Excision radical, large intestine |
| **CCI Codes for Colectomy in Ulcerative Colitis** | |
| 1NM87.x | Excision partial, large intestine |
| 1NM89.x | Excision total, large intestine |
| 1NQ89.x | Excision total, rectum (including proctocolectomy) |
| 1NQ90.x | Excision total with reconstruction, rectum |
| 1NM91.x | Excision radical, large intestine |

*Was not included in validation algorithm but was used prior to 2006 and was replaced by 1NQ89

**Was not included in the CD validation algorithm but was included in the UC validation algorithm

**Supplemental Table 3. Differences in life expectancy at 65 years comparing medications used to treat Crohn’s disease in seniors. Differences correspond to the medication referenced in the column name subtracted from the medication reference in the row name (i.e., LE­_row_ – LE_column_). Significant differences are indicated in bold font.**

|  |  | **Mesalamine** | **Immunomodulator Monotherapy** | **Biologic Monotherapy** | **Combination Therapy** | **Systemic Steroids** |
| --- | --- | --- | --- | --- | --- | --- |
| No Therapy | Females | **-3.1 (-3.9, -2.3)** | **2.9 (1.8, 4.1)** | 0.9 (-1.9, 3.8) | **-3.9 (-7.2, -0.5)** | **6.4 (5.4, 7.4)** |
|  | Males | **-2.3 (-3.1, -1.4)** | **3.3 (2.1, 4.5)** | **3.9 (2.0, 5.9)** | 0.0 (-3.8, 3.8) | **6.5 (5.5, 7.5)** |
| Mesalamine | Females |  | **6.0 (4.8, 7.3)** | **4.0 (1.2, 6.9)** | -0.8 (-4.1, 2.6) | **9.5 (8.4, 10.6)** |
|  | Males |  | **5.6 (4.3, 6.8)** | **6.2 (4.2, 8.2)** | 2.3 (-1.6, 6.1) | **8.8 (7.7, 9.9)** |
| Immunomodulator Monotherapy | Females |  |  | -2.0 (-5.0, 1.0) | **-6.8 (-10.3, -3.3)** | **3.5 (2.1, 4.9)** |
|  | Males |  |  | 0.6 (-1.5, 2.8) | -3.3 (-7.2, 0.6) | **3.2 (1.8, 4.6)** |
| Biologic Monotherapy | Females |  |  |  | **-4.8 (-9.1, -0.5)** | **5.5 (2.5, 8.4)** |
|  | Males |  |  |  | -4.0 (-8.2, 0.3) | **2.6 (0.5, 4.6)** |
| Combination Therapy | Females |  |  |  |  | **10.3 (6.8, 13.7)** |
|  | Males |  |  |  |  | **6.5 (2.6, 10.4)** |

**Supplemental Table 4. Differences in life expectancy at 65 years comparing medications used to treat ulcerative colitis in seniors. Differences correspond to the medication referenced in the column name subtracted from the medication reference in the row name (i.e., LE­_row_ – LE_column_). Significant differences are indicated in bold font.**

|  |  | **Mesalamine** | **Immunomodulator Monotherapy** | **Biologic Monotherapy** | **Combination Therapy** | **Systemic Steroids** |
| --- | --- | --- | --- | --- | --- | --- |
| No Therapy | Females | **-3.4 (-4.1, -2.8)** | **3.3 (1.9, 4.6)** | **4.1 (1.4, 6.7)** | 3.4 (-1.5, 8.4) | **7.2 (6.1, 8.3)** |
|  | Males | **-2.7 (-3.3, -2.1)** | **3.4 (2.4, 4.3)** | -0.4 (-3.6, 2.9) | 1.9 (-1.1, 5.0) | **7.0 (6.2, 7.9)** |
| Mesalamine | Females |  | **6.7 (5.3, 8.0)** | **7.5 (4.8, 10.2)** | **6.9 (1.9, 11.8)** | **10.6 (9.4, 11.7)** |
|  | Males |  | **6.1 (5.1, 7.1)** | 2.3 (-0.9, 5.6) | **4.6 (1.6, 7.7)** | **9.7 (8.8, 10.6)** |
| Immunomodulator Monotherapy | Females |  |  | 0.8 (-2.1, 3.7) | 0.2 (-4.8, 5.2) | **3.9 (2.3, 5.5)** |
|  | Males |  |  | **-3.7 (-7.1, -0.3)** | -1.4 (-4.6, 1.8) | **3.6 (2.5, 4.8)** |
| Biologic Monotherapy | Females |  |  |  | -0.6 (-6.2, 4.9) | **3.1 (0.3, 5.9)** |
|  | Males |  |  |  | 2.3 (-2.1, 6.7) | **7.4 (4.0, 10.7)** |
| Combination Therapy | Females |  |  |  |  | 3.7 (-1.3, 8.7) |
|  | Males |  |  |  |  | **5.1 (1.9, 8.2)** |

**Supplemental Table 5. Differences in life expectancy at 65 years comparing medications used to treat Crohn’s disease in seniors, censoring at intestinal resection or colectomy. Differences correspond to the medication referenced in the column name subtracted from the medication reference in the row name (i.e., LE­_row_ – LE_column_). Significant differences are indicated in bold font.**

|  |  | **Mesalamine** | **Immunomodulator Monotherapy** | **Biologic Monotherapy** | **Combination Therapy** | **Systemic Steroids** |
| --- | --- | --- | --- | --- | --- | --- |
| **No Therapy** | Females | **-3.8 (-4.6, -3.0)** | **2.4 (1.2, 3.6)** | 1.0 (-2.0, 4.0) | -3.3 (-6.8, 0.1) | **5.7 (4.7, 6.7)** |
|  | Males | **-3.2 (-4.1, -2.4)** | **2.6 (1.3, 3.8)** | **15.2 (13.6, 16.8)** | 0.5 (-3.5, 4.5) | **5.2 (4.2, 6.2)** |
| **Mesalamine** | Females |  | **6.2 (4.9, 7.5)** | **4.8 (1.7, 7.8)** | 0.5 (-3.0, 4.0) | **9.5 (8.4, 10.7)** |
|  | Males |  | **5.8 (4.5, 7.1)** | **18.4 (16.8, 20.1)** | 3.7 (-0.3, 7.7) | **8.4 (7.3, 9.5)** |
| **Immunomodulator Monotherapy** | Females |  |  | -1.4 (-4.6, 1.7) | **-5.7 (-9.3, -2.1)** | **3.3 (1.9, 4.8)** |
|  | Males |  |  | **12.7 (10.8, 14.6)** | -2.1 (-6.2, 2.0) | **2.6 (1.2, 4.0)** |
| **Biologic Monotherapy** | Females |  |  |  | -4.3 (-8.8, 0.3) | **4.8 (1.6, 7.9)** |
|  | Males |  |  |  | **-14.8 (-19.0, -10.5)** | **-10.1 (-11.8, -8.3)** |
| **Combination Therapy** | Females |  |  |  |  | **9 (5.5, 12.6)** |
|  | Males |  |  |  |  | **4.7 (0.6, 8.8)** |

**Supplemental Table 6. Differences in life expectancy at 65 years comparing medications used to treat ulcerative colitis in seniors, censoring at colectomy. Differences correspond to the medication referenced in the column name subtracted from the medication reference in the row name (i.e., LE­_row_ – LE_column_). Significant differences are indicated in bold font.**

|  |  | **Mesalamine** | **Immunomodulator Monotherapy** | **Biologic Monotherapy** | **Combination Therapy** | **Systemic Steroids** |
| --- | --- | --- | --- | --- | --- | --- |
| **No Therapy** | Females | **-3.4 (-4.1, -2.8)** | **3.3 (2.0, 4.6)** | **3.4 (0.4, 6.5)** | 3.4 (-1.5, 8.3) | **7.2 (6.1, 8.3)** |
|  | Males | **-2.7 (-3.3, -2.1)** | **3.4 (2.4, 4.4)** | -0.6 (-3.9, 2.6) | 1.3 (-1.8, 4.5) | **7.0 (6.2, 7.9)** |
| **Mesalamine** | Females |  | **6.7 (5.4, 8.1)** | **6.9 (3.8, 9.9)** | **6.9 (2, 11.8)** | **10.6 (9.5, 11.8)** |
|  | Males |  | **6.1 (5.1, 7.1)** | 2.1 (-1.2, 5.3) | **4.0 (0.9, 7.2)** | **9.7 (8.8, 10.6)** |
| **Immunomodulator Monotherapy** | Females |  |  | 0.1 (-3.1, 3.4) | 0.1 (-4.9, 5.2) | **3.9 (2.3, 5.5)** |
|  | Males |  |  | **-4.0 (-7.3, -0.7)** | -2.0 (-5.3, 1.2) | **3.6 (2.5, 4.8)** |
| **Biologic Monotherapy** | Females |  |  |  | 0 (-5.7, 5.7) | **3.8 (0.6, 7.0)** |
|  | Males |  |  |  | 2.0 (-2.5, 6.4) | **7.7 (4.3, 11.0)** |
| **Combination Therapy** | Females |  |  |  |  | 3.8 (-1.2, 8.8) |
|  | Males |  |  |  |  | **5.7 (2.5, 8.9)** |

**Supplemental Figure 1. Life expectancy at 65 years of age in (A) females and (B) males with Crohn’s disease, stratified by type of medical treatment.**

1. **Females**


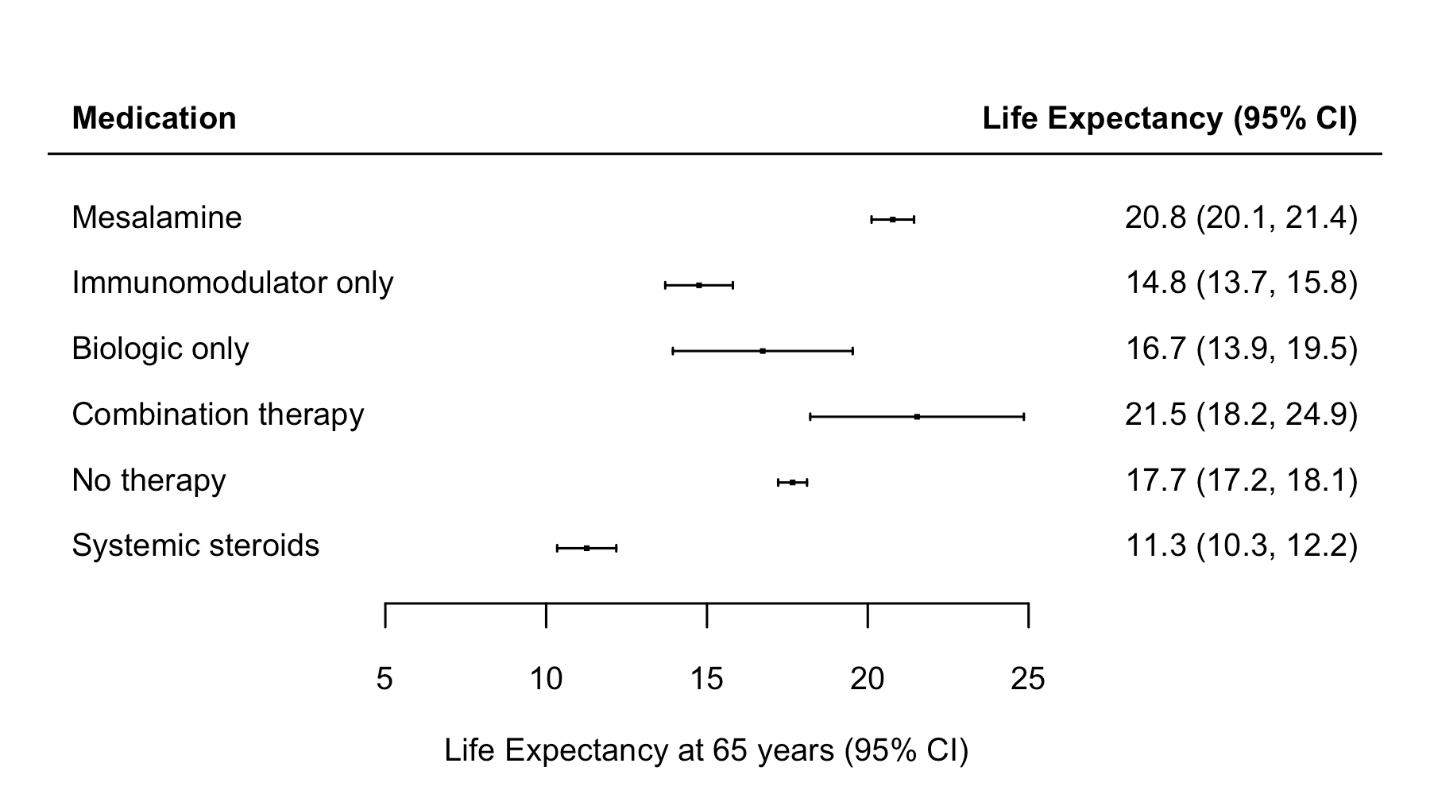


1. **Males**


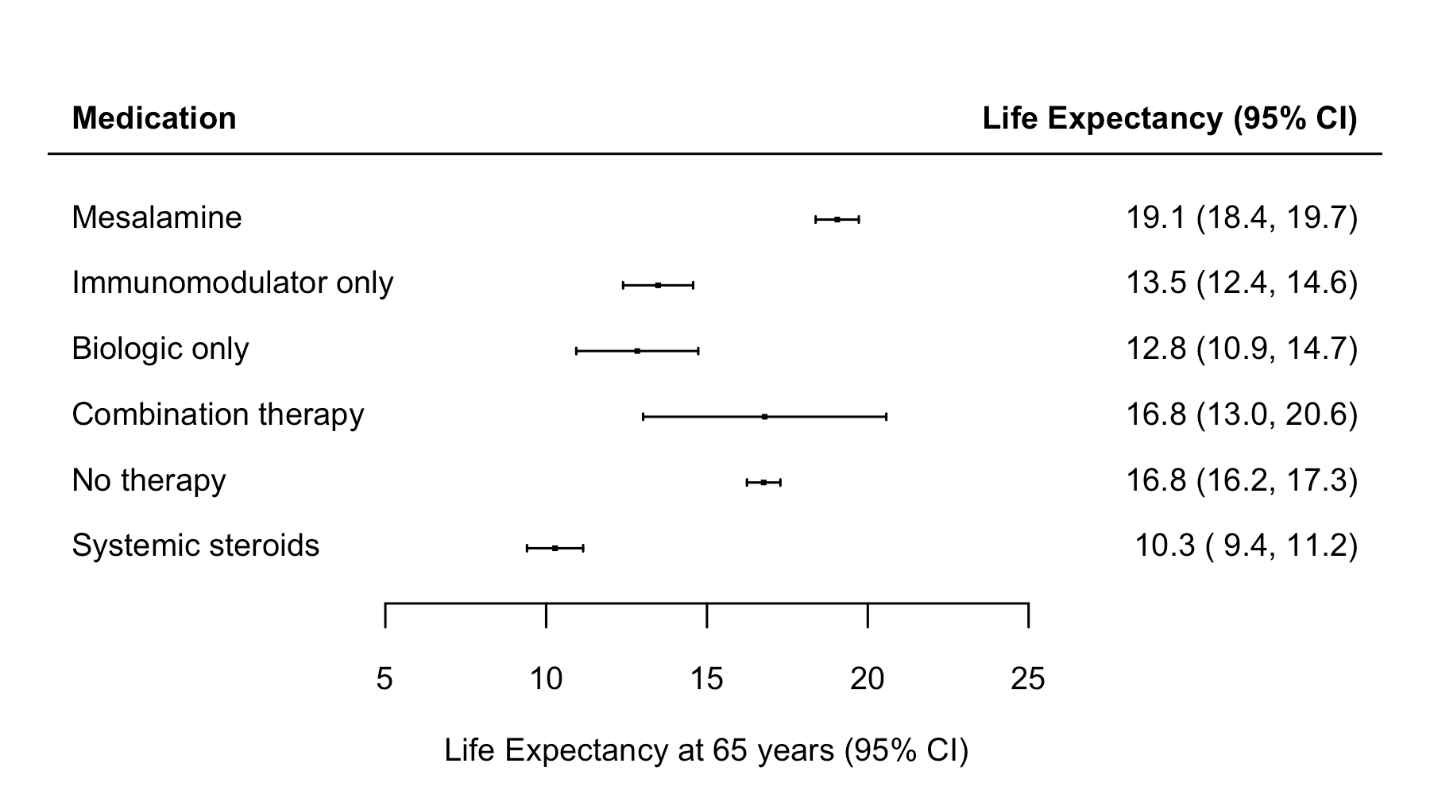


**Supplemental Figure 2. The proportion of people expected to be alive at each age interval based on type of medical treatment in (A) females and (B) males with Crohn’s disease.**

**
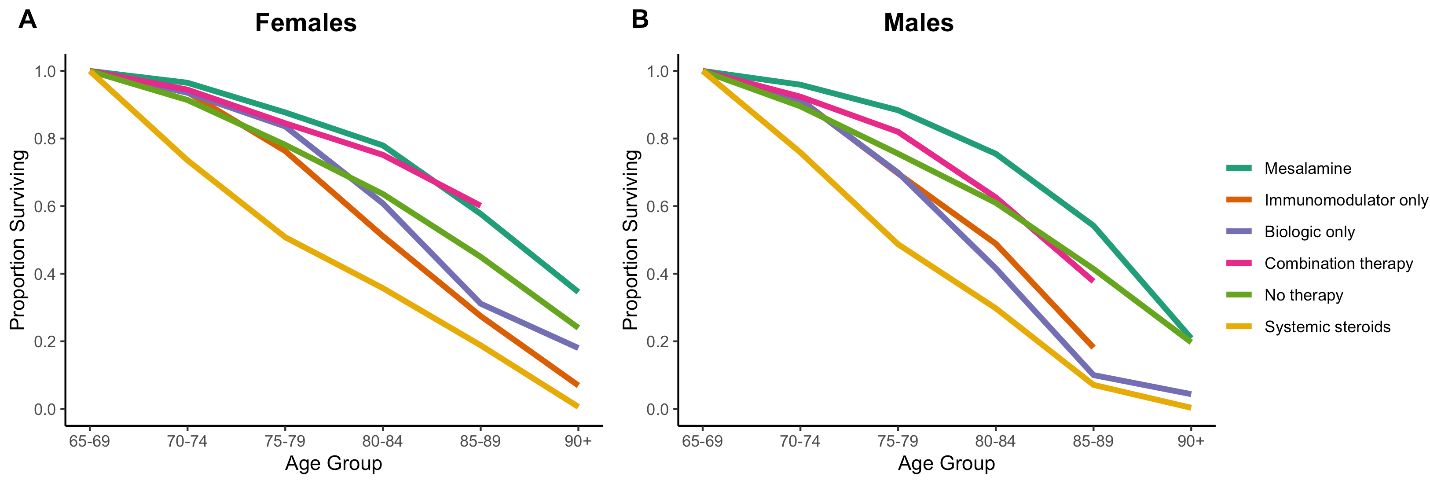
**

**Supplemental Figure 3. Life expectancy at 65 years of age in (A) females and (B) males with ulcerative colitis, stratified by type of medical treatment.**

1. **Females**


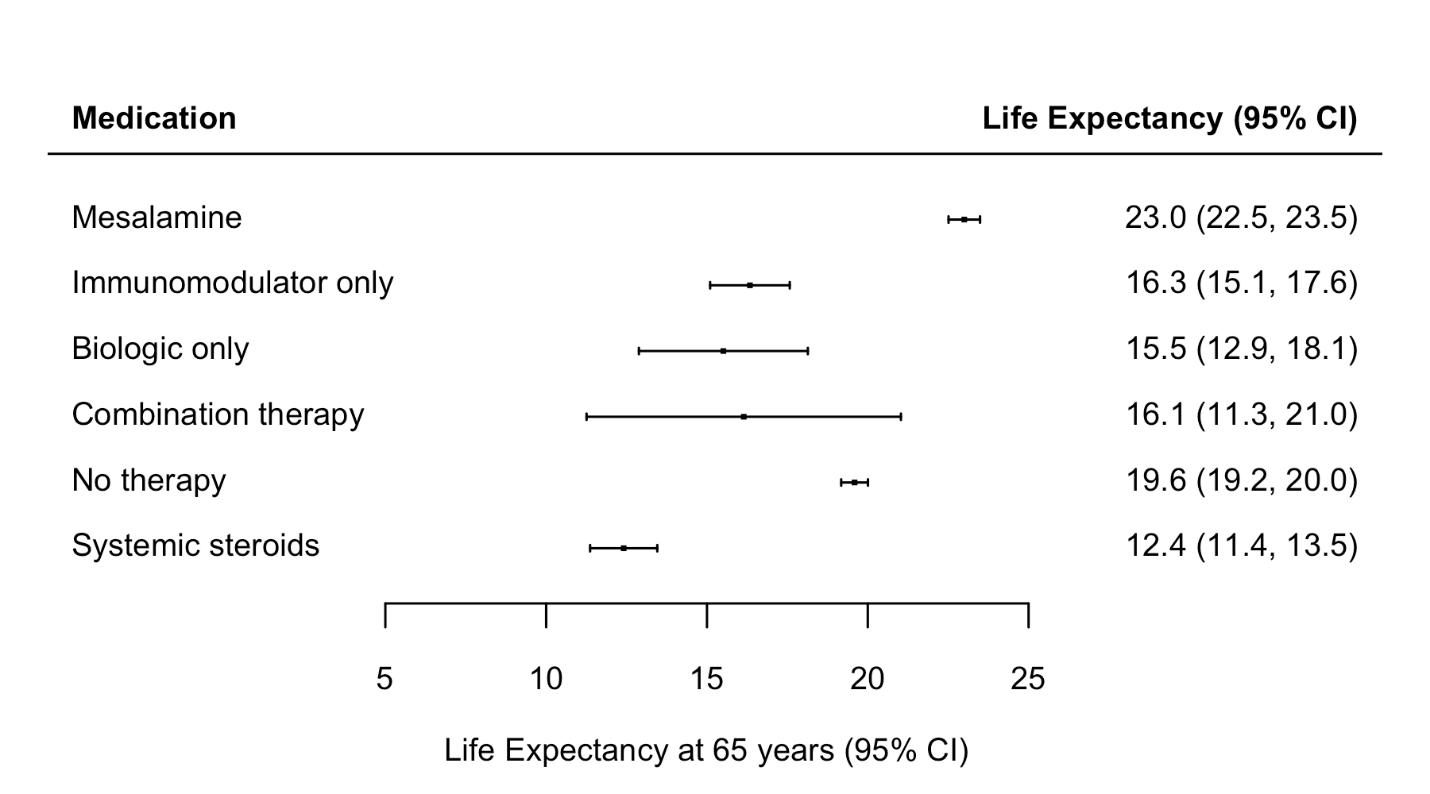


1. **Males**


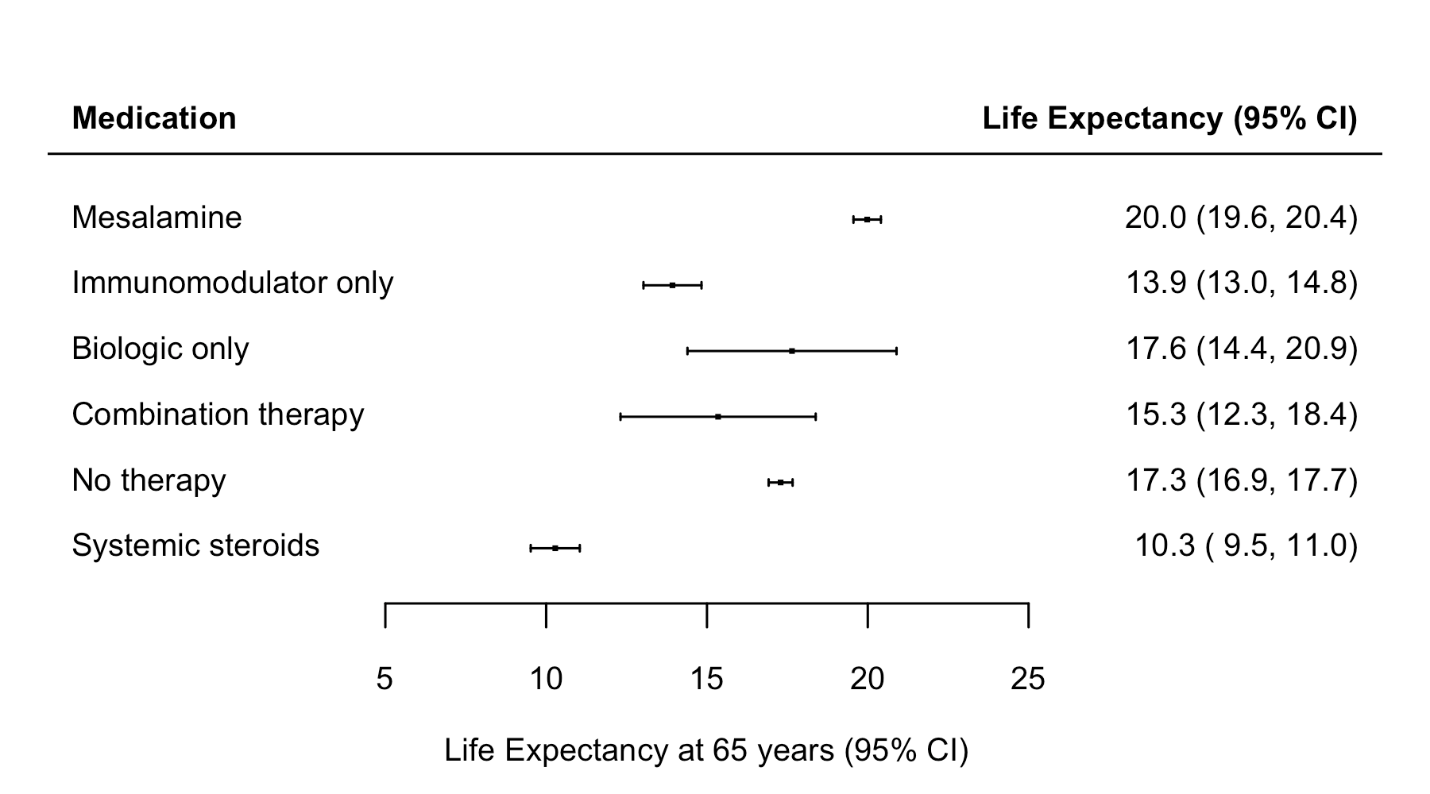


**Supplemental Figure 4. The proportion of people expected to be alive at each age interval based on type of medical treatment in (A) females and (B) males with ulcerative colitis.**

**
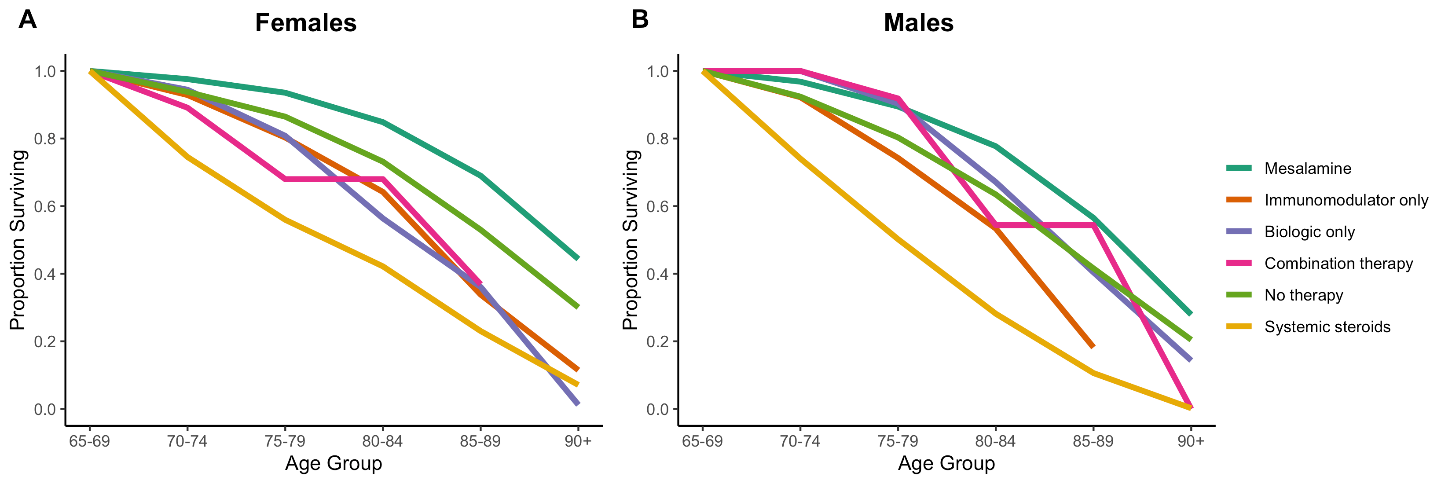
**

**Supplemental Figure 5. Life expectancy at 65 years of age in (A) females and (B) males with Crohn’s disease, stratified by type of medical treatment and censored at intestinal resection or surgery.**

1. **Females**


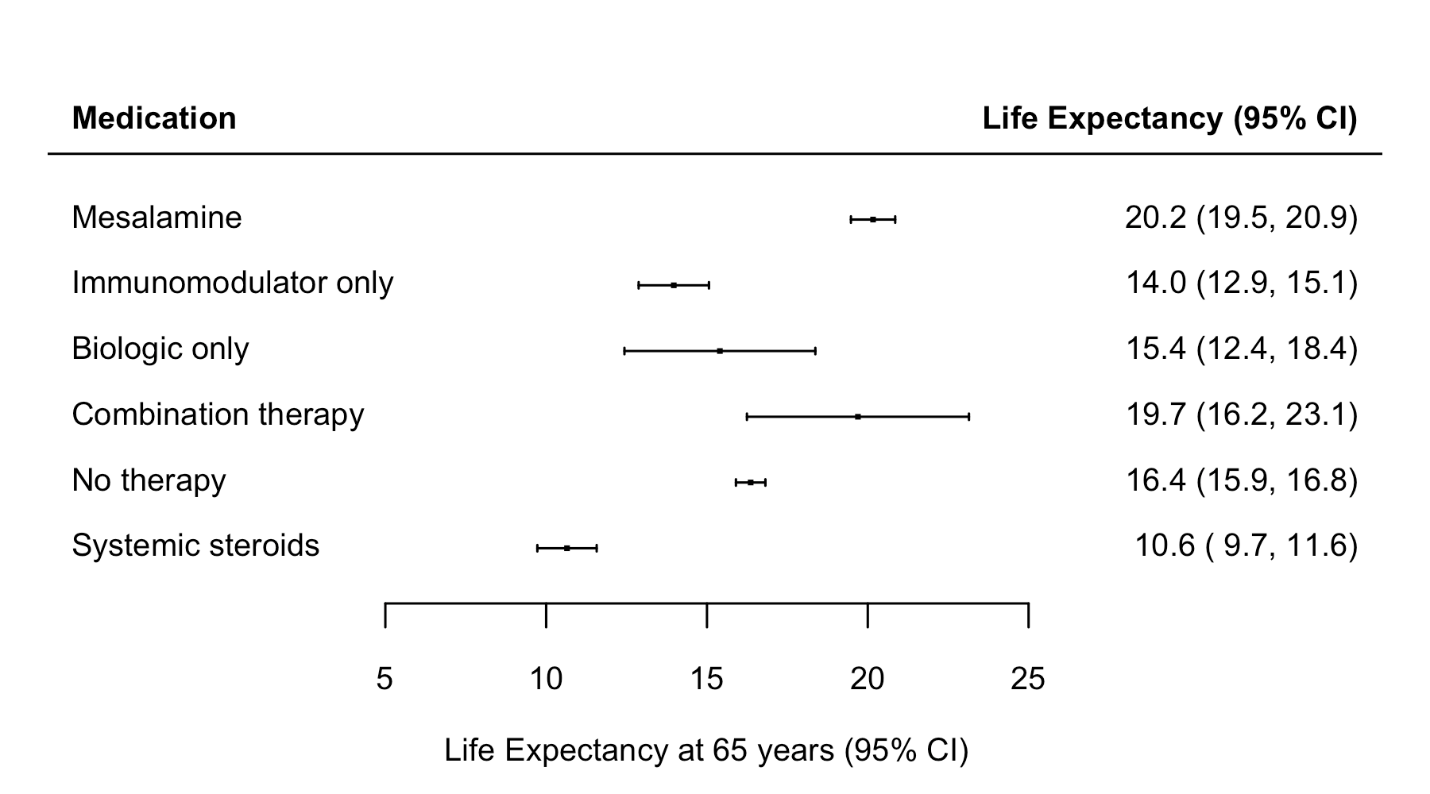


1. **Males**


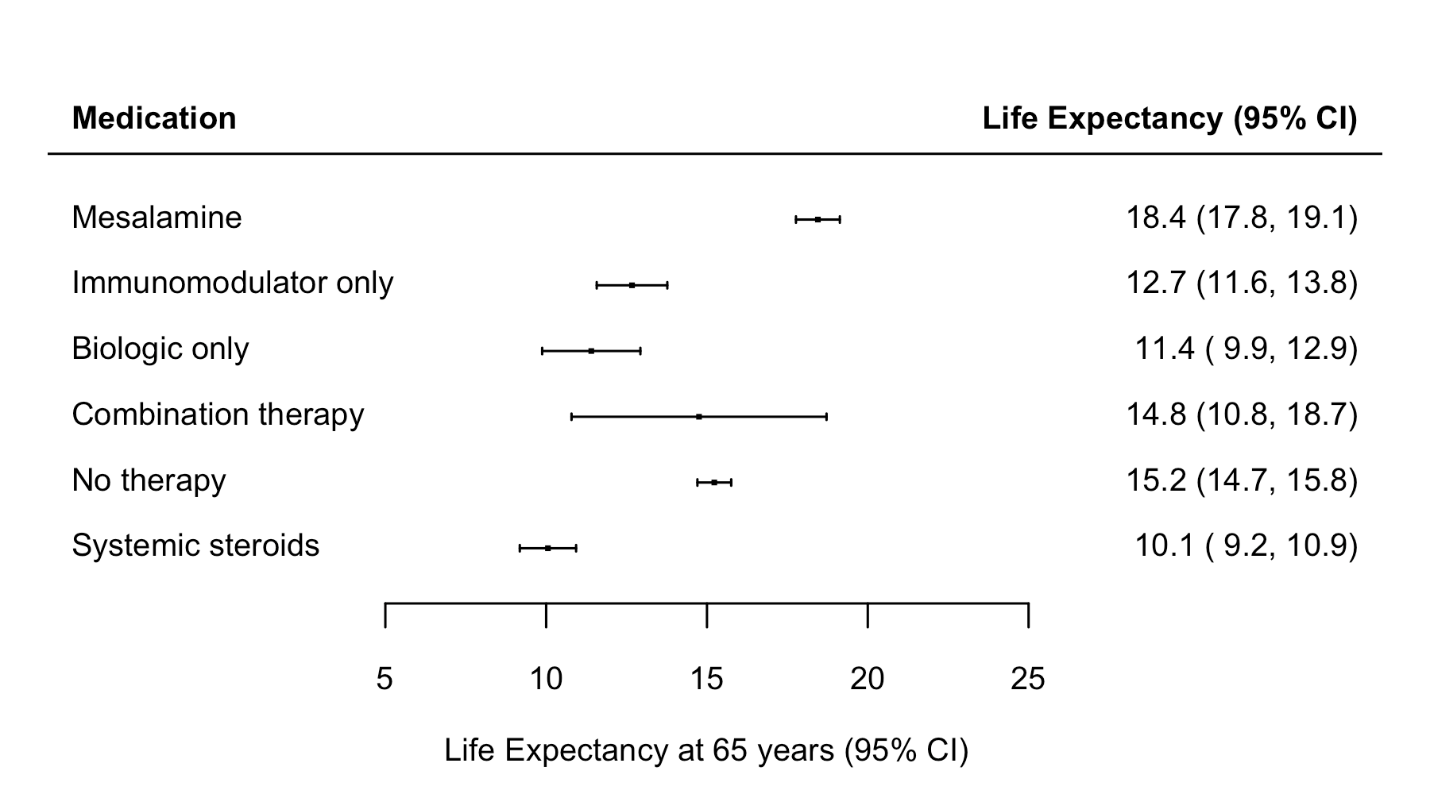


**Supplemental Figure 6. Life expectancy at 65 years of age in (A) females and (B) males with ulcerative colitis, stratified by type of medical treatment and censored at colectomy.**

1. **Females**


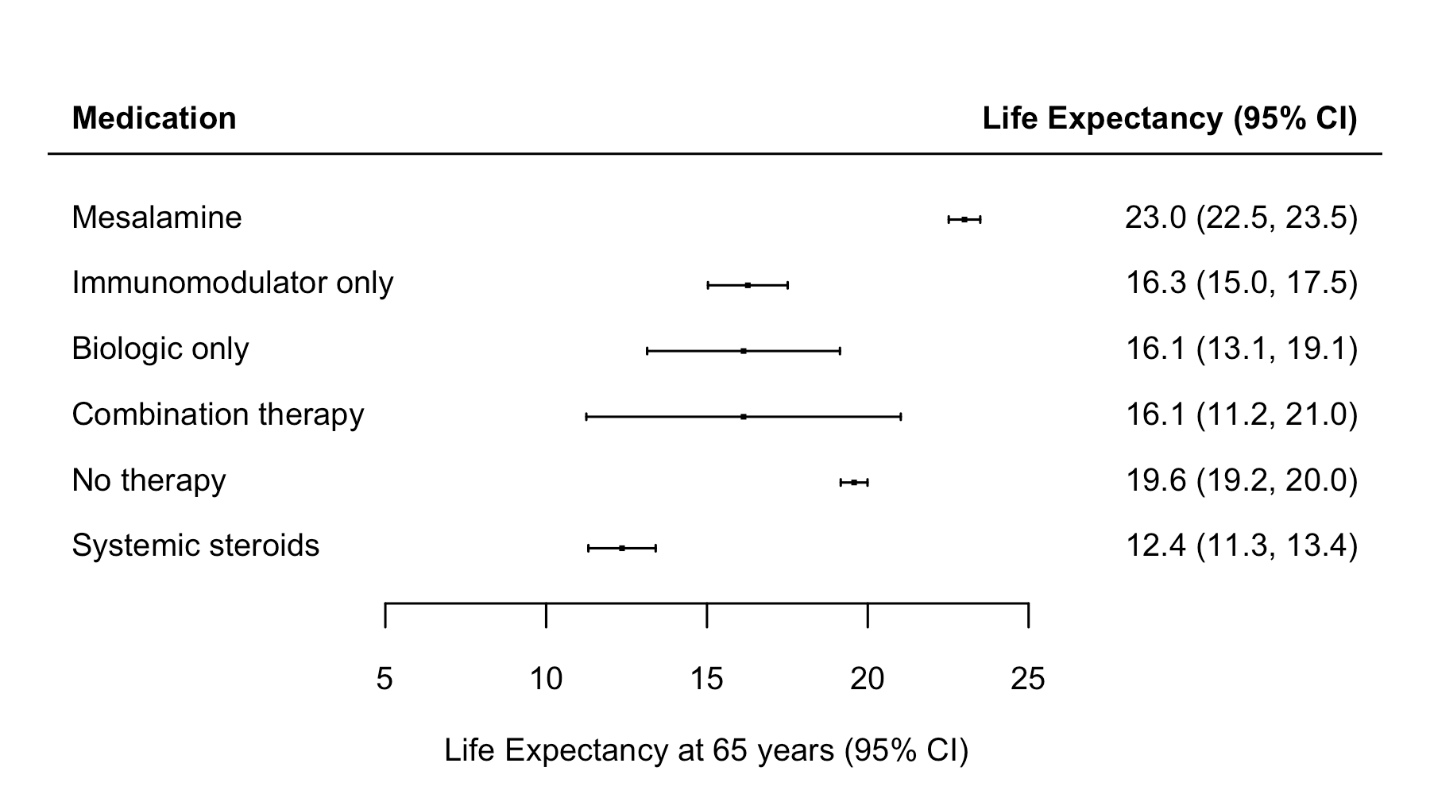


1. **Males**


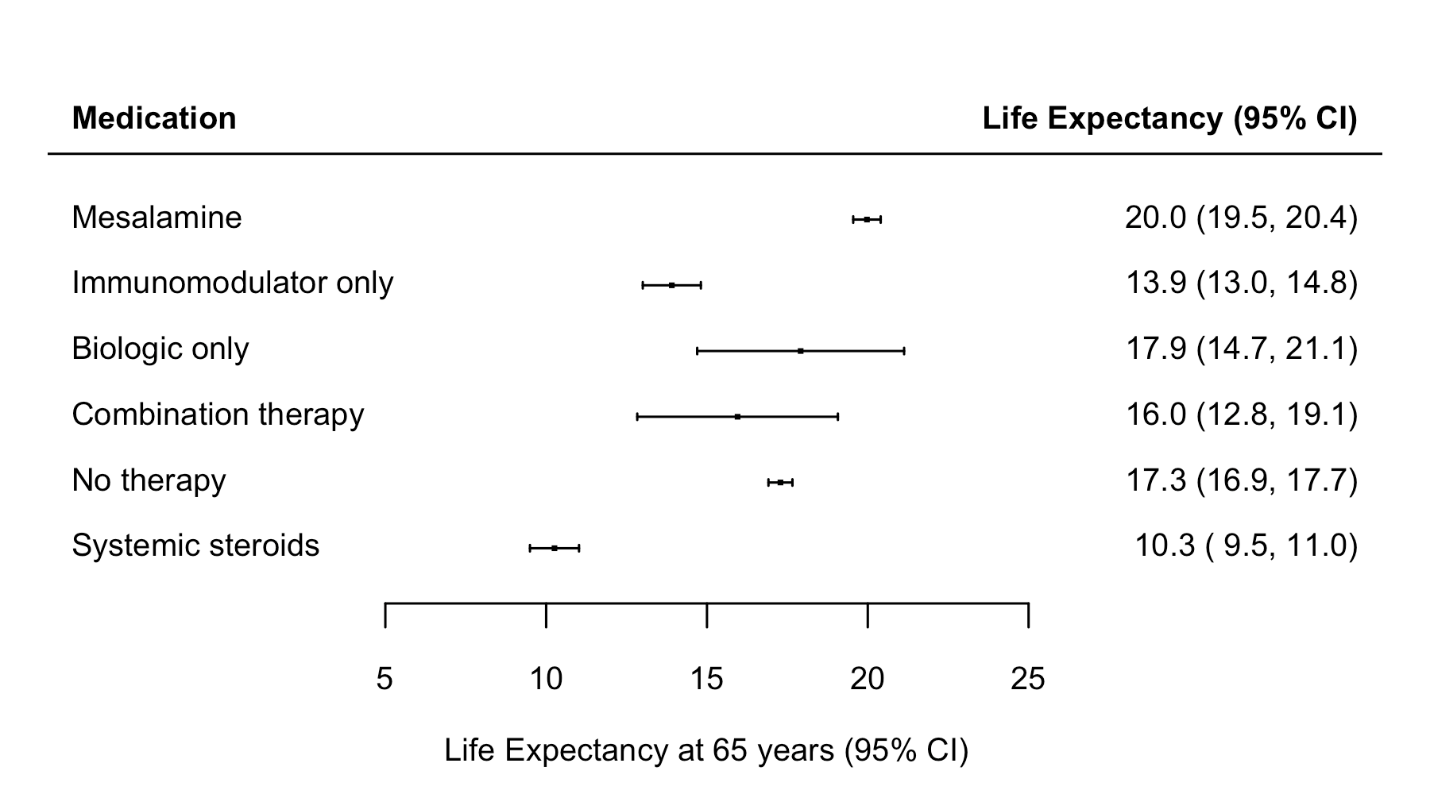


**Supplemental Figure 7. The proportion of people expected to be alive at each age interval based on type of medical treatment in (A) females and (B) males with Crohn’s disease, censored at intestinal resection or colectomy.**


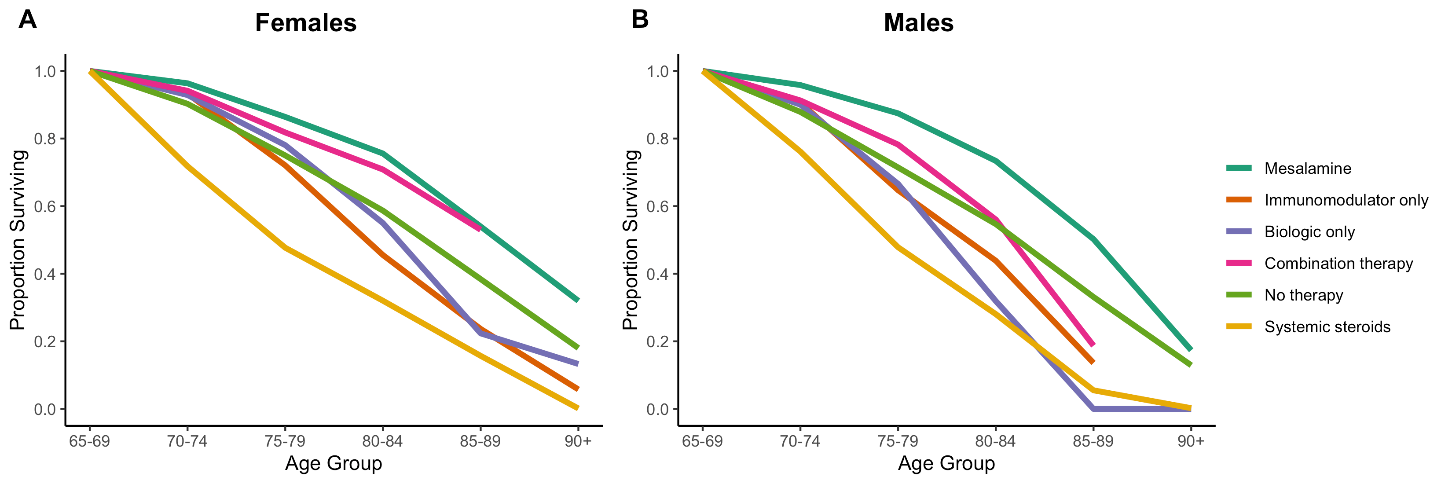


**Supplemental Figure 8. The proportion of people expected to be alive at each age interval based on type of medical treatment in (A) females and (B) males with ulcerative colitis, censoring at colectomy.**


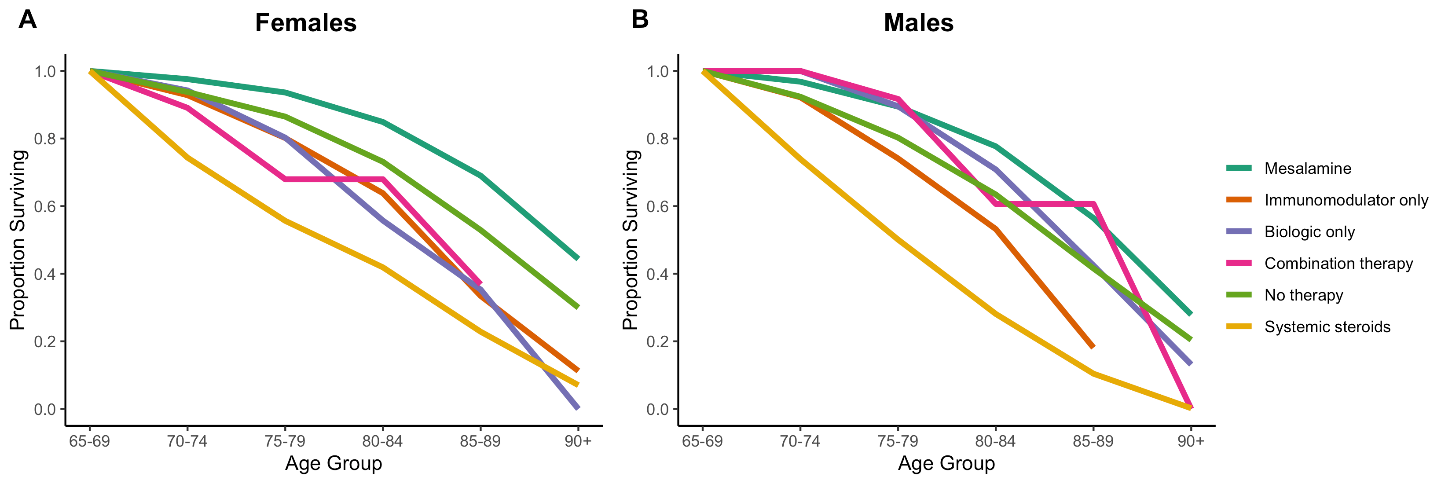

Supplement: Supplementary file 1 — Additional file 1. Table S1. List of drug identification numbers used to identify medications included in this study. Table S2. List of validated codes used to identify patients requiring small bowel resection (Crohn’s disease) or colectomy (Crohn’s disease and ulcerative colitis). Table S3. Differences in life expectancy at 65 years comparing medications used to treat Crohn’s disease in seniors. Differences correspond to the medication referenced in the column name subtracted from the medication reference in the row name (i.e., LErow – LEcolumn). Significant differences are indicated in bold font. Table S4. Differences in life expectancy at 65 years comparing medications used to treat ulcerative colitis in seniors. Differences correspond to the medication referenced in the column name subtracted from the medication reference in the row name (i.e., LErow – LEcolumn). Significant differences are indicated in bold font. Table S5. Differences in life expectancy at 65 years comparing medications used to treat Crohn’s disease in seniors, censoring at intestinal resection or colectomy. Differences correspond to the medication referenced in the column name subtracted from the medication reference in the row name (i.e., LErow – LEcolumn). Significant differences are indicated in bold font. Table S6. Differences in life expectancy at 65 years comparing medications used to treat ulcerative colitis in seniors, censoring at colectomy. Differences correspond to the medication referenced in the column name subtracted from the medication reference in the row name (i.e., LErow – LEcolumn). Significant differences are indicated in bold font. Figure S1. Life expectancy at 65 years of age in (A) females and (B) males with Crohn’s disease, stratified by type of medical treatment. Figure S2. The proportion of people expected to be alive at each age interval based on type of medical treatment in (A) females and (B) males with Crohn’s disease. Figure S3. Life expectancy at 65 years of age [file 12876_2021_2083_MOESM1_ESM.docx]
